# Cruroplasty as a standalone treatment for recurrent hiatal hernia repair

**DOI:** 10.1007/s10029-024-03088-8

**Published:** 2024-06-19

**Authors:** Ashley Tran, Luke R. Putnam, Lucy Harvey, John C. Lipham

**Affiliations:** Division of Upper GI and General Surgery, Keck School of Medicine of USC, 1510 San Pablo St. #514, Los Angeles, CA 90033 USA

**Keywords:** Hiatal hernia, Cruroplasty, Fundoplication, Antireflux surgery

## Abstract

**Purpose:**

Following laparoscopic anti-reflux surgery (LARS), recurrence of hiatal hernia is common. Patients with symptomatic recurrence typically undergo revision of the fundoplication or conversion to magnetic sphincter augmentation (MSA) in addition to cruroplasty. However, patients with an intact fundoplication or MSA may only require repeat cruroplasty to repair their recurrent hiatal hernia. The purpose of this study is to compare outcomes following cruroplasty alone compared to full revision (i.e. redo fundoplication or MSA with cruroplasty) for the management of recurrent hiatal hernias.

**Methods:**

A retrospective review of patients undergoing surgical revision of a symptomatic recurrent hiatal hernia between February 2009 and October 2022 was performed. Preoperative characteristics, intraoperative details, and postoperative outcomes were compared between patients undergoing cruroplasty alone versus full revision.

**Results:**

A total of 141 patients were included in the analysis. 93 patients underwent full revision, and 48 patients underwent cruroplasty alone. The mean time between initial and revisional surgery was 8 ± 7.7 years. There was no significant difference in operative time or rates of intra-operative or post-operative complication between groups. Patients undergoing cruroplasty alone had a mean Gastroesophageal Reflux Disease Health Related Quality Life (GERD-HRQL) Questionnaire score of 9.6 ± 10.2 compared to a mean score of 8.9 ± 11.2 for full revision patients (p = 0.829). Recurrence rates following revision was 10.4% for cruroplasty alone patients and 11.8% in full revision patients (p > 0.999).

**Conclusion:**

In patients with intact fundoplication or MSA, cruroplasty alone results in similar post-operative outcomes compared to full revision for recurrent hiatal hernia.

## Introduction

Gastroesophageal reflux disease (GERD) is a common disorder affecting nearly a third of the United States population [1]. There is a well-recognized association between hiatal hernias and GERD [2]. The mechanism behind the development of GERD in patients with hiatal hernias is related to reduced lower esophageal sphincter pressure and disruption of the diaphragmatic crura [2, 3]. Laparoscopic repair of hiatal hernias, typically involving reduction of the hernia sac, crural closure, and fundoplication, has demonstrated excellent results in terms of symptom relief and improved quality of life [4, 5]. Following laparoscopic repair of large hiatal hernias, recurrence rates as high as 60% have been reported [6]. The causes of recurrence are multifactorial and may include failure to achieve sufficient intra-abdominal esophageal length, inadequate crural closure, preexisting medical conditions, and the size of the hiatal hernia [4, 7].

Despite high recurrence rates, many patients have mild to moderate symptoms which are managed medically [4]. Patients with significant or persistent symptoms or large recurrent HHs often require additional surgery [4, 8]. Re-operative HH procedures, most commonly involving redo cruroplasty and fundoplication, are technically difficult due to adhesions and distorted anatomy and have been associated with intraoperative morbidity rates of nearly 20%, mainly from gastric and esophageal perforations [9–11].

Given the significant morbidity associated with redo operations and the theory that most of the reflux in these patients is due to recurrent hiatal hernias, we set out to determine whether or not redoing the fundoplication was necessary or not. In other words, based on evidence highlighting the significant role of the diaphragmatic crura in the GERD barrier following laparoscopic anti-reflux surgery (LARS), cruroplasty alone for management of recurrent hiatal hernias in patients with intact fundoplications may decrease the morbidity associated with these operations while reestablishing an effective GERD barrier [12]. This study aims to expand on prior research to further evaluate outcomes following cruroplasty alone compared to cruroplasty and fundoplication revision for the management of recurrent hiatal hernias.

## Materials and methods

### Patient selection

Following IRB approval, a retrospective review of patients with HH recurrence following LARS, defined as cruroplasty and fundoplication or magnetic sphincter augmentation (MSA), was performed. Adult patients (age > 18 years) who underwent surgical repair of a recurrent HH after LARS between February 2009 and October 2022 at two tertiary medical centers were included. Revisional surgeries included redo fundoplication or MSA revision/replacement or cruroplasty alone with or without mesh.

### Pre-operative evaluation

Patient demographics including age, gender, and body mass index (BMI) were recorded. Information regarding presenting symptoms, such as heartburn, dysphagia, nausea and vomiting, duration of GERD symptoms, and acid suppression medication use was also recorded. Symptom severity was assessed using the GERD-Health Related Quality of Life (HRQL) survey [13], a questionnaire comprised of ten questions regarding typical symptoms of GERD. Each question is scored from 0 to 5 for a total score ranging from 0 to 50. Higher scores correlate with more severe symptoms. The initial type of LARS was recorded and the time between the initial and revisional LARS was determined.

Pre-operative testing included an EGD, during which hiatal hernia size, Hill Grade, and the presence of Barrett’s esophagus was recorded. A videoesophagram (VEG) to determine the size of the recurrent hiatal hernia. Recurrent hiatal hernia was defined as the presence of a hiatal hernia greater than 2 cm on VEG or during EGD [14] in a patient who previously underwent LARS.

### Operative characteristics

Cruroplasty with or without mesh was performed according to the technique described by Nguyen et al. [12]. Following hiatal hernia repair, intraoperative endoscopy was performed. If the original fundoplication or MSA device appeared intact (i.e. Hill Grade of I), it was left in place. Otherwise, the prior revision was taken down and redone or converted to either MSA or fundoplication. Intraoperative data such as hiatal hernia size, type of LARS performed, use of mesh and mesh type, operative time, and intraoperative complications was recorded. Intraoperative esophagogastroduodenoscopy was performed following repair to confirm satisfactory hernia reduction and valve integrity.

### Post-operative follow-up

Post-operative data regarding acid suppression medication use, GERD-HRQL score, 30-day complications, additional interventions, including dilation and reoperation, readmission, or emergency department (ED) visits was collected. Data from follow-up EGD and VEG, including HH recurrence, was recorded.

### Statistical analysis

Univariate analysis was performed using Chi-square or Fisher’s exact tests to analyze categorical variables and student t-test or Wilcoxon Ranked Sum tests for continuous variables. A p-value < 0.05 was considered statistically significant. SPSS version 29 was used for all statistical analysis.

## Results

### Pre-operative characteristics

During the study period, a total of 145 patients underwent revisional surgery for recurrent HH. Four patients underwent a revision other than redo fundoplication, LINX revision or placement, or cruroplasty alone (ex. Roux-en-Y gastric bypass) and were excluded from analysis. Of the remaining 141 patients, 56.0% were female, with a mean age of 66 (± 14.5) years and a mean BMI of 27.9 (± 6.0). Forty-seven patients (33.3%) had GERD symptoms for greater than 10 years. The most commonly cited preoperative symptoms were heartburn (n = 80, 56.7%), followed by regurgitation (n = 44, 31.2%), and dysphagia (n = 41, 29.1%). At the time of initial assessment, 93 patients (66.0%) were taking proton pump inhibitors. The mean time between the initial and revisional LARS was 8.0 (± 7.7) years.

93 patients (66.0%) underwent a full revision (i.e. Cruroplasty and fundoplication or LINX) and 48 patients (34.0%) underwent cruroplasty alone, with or without mesh. Compared to the cruroplasty alone patients, patients receiving full revision had smaller recurrent HH size (4.5 ± 2.3 vs 5.8 ± 2.9 cm, p = 0.049). Additionally, there were more patients in the full revision group who had evidence of Barret’s esophagus found on preoperative EGD (29.2% vs 5.9%, p = 0.023). There were no significant differences between the two groups in the remaining preoperative characteristics (Table 1).

**Table 1:** Comparison of pre-operative characteristics between the full revision and cruroplasty alone groups

	Full revision (n = 93)	Cruroplasty alone (n = 48)	p-value
Age	64.9 ± 13.1	69.0 ± 16.7	0.117
Gender (Female)	49 (52.7%)	30 (62.5%)	0.288
BMI	27.2 ± 5.6	29.7 ± 6.8	0.071
Duration of GERD Symptoms			0.565
< 1 year	4 (6.0%)	3 (10.3%)	
1–5 years	21 (31.3%)	6 (20.7%)	
6–9 years	9 (13.4%)	6 (20.7%)	
> 10 years	33 (49.3%)	14 (48.3%)	
Years to revisional surgery	7.1 ± 7.8	9.2 ± 7.6	0.163
Preop PPI	61 (65.6%)	32 (66.7%)	0.600
Preop dysphagia	26 (35.6%)	15 (44.1%)	0.404
Preop heartburn	51 (71.8%)	29 (80.6%)	0.358
Preop regurgitation	29 (40.3%)	15 (46.9%)	0.668
Preop cough	14 (20.0%)	9 (25.7%)	0.618
Preop hoarseness	14 (20.0%)	7 (21.9%)	0.799
Preop HH size (cm)	4.5 ± 2.3	5.8 ± 2.9	0.049*
Preop LA Esophagitis Class			0.511
A	6 (8.7%)	2 (7.1%)	
B	10 (14.5%)	1 (3.6%)	
C	6 (8.7%)	2 (7.1%)	
D	0 (0.0%)	1 (1.4%)	
Preop Hill Grade			0.838
1	12 (33.3%)	3 (23.1%)	
2	3 (8.3%)	2 (15.4%)	
3	6 (16.7%)	2 (15.4%)	
4	15 (41.7%)	6 (46.2%)	
Barrett's Esophagus classification			0.023*
None	46 (70.8%)	32 (94.1%)	
Short	14 (21.5%)	2 (5.9%)	
Long	5 (7.7%)	0 (0.0%)	
Preop GERD HRQL Score	23.8 ± 12.2	23.3 ± 16.8	0.926

Continuous data expressed as mean ± standard deviation; categorical data represented as n (%)

^*^p < 0.005

*BMI* Body mass index, *GERD* Gastroesophageal reflux disease, *PPI* Proton pump inhibitor use, *HH* Hiatal hernia

### Intra-operative characteristics

**Fig. 1:**
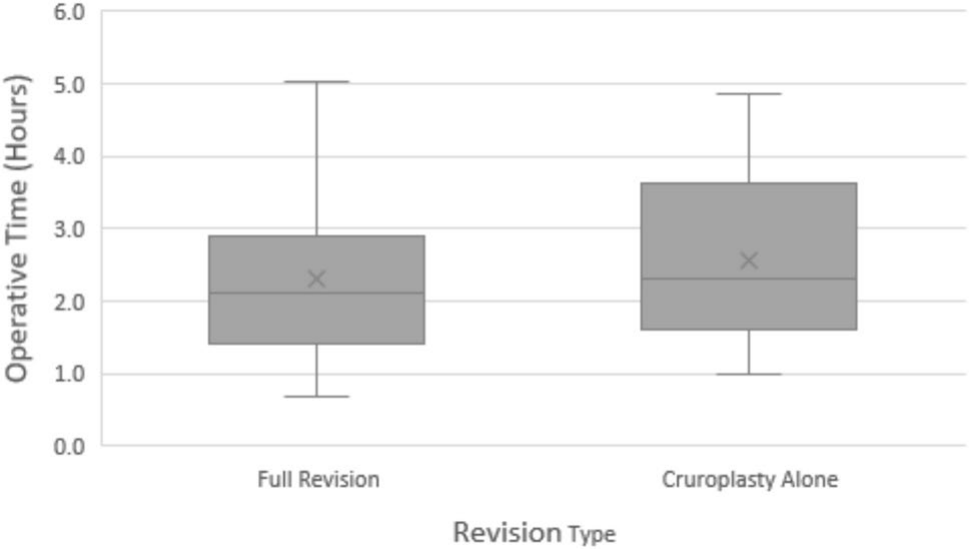
Comparison of median operative time between full revision and cruroplasty alone

**Fig. 2:**
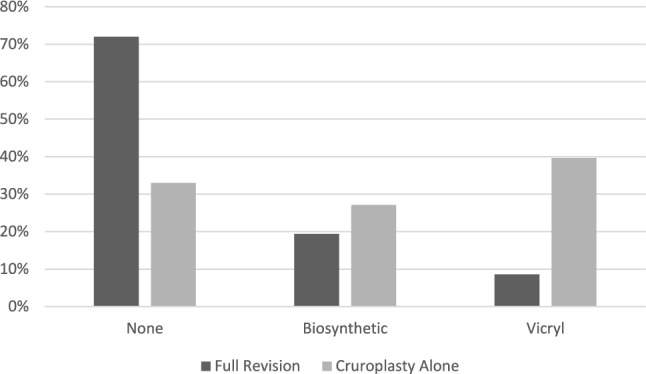
Comparison of mesh use during full revision or cruroplasty alone

A comparison of intra-operative characteristics between the two groups is summarized in Table 2. The median operative time (Fig. 1) for the full revision group was comparable to that of the cruroplasty alone group (129 vs 138 min, p = 0.349). Placement of mesh (Fig. 2) was more common in patients who received cruroplasty alone (66.7% vs 28.0%, p < 0.001). Among the patients who underwent repair with mesh, 21 patients received vicryl mesh (full revision: n = 8, 8.6%; cruroplasty: n = 13, 27.1%, p = 0.010) and 37 patients received biosynthetic mesh (full revision: n = 18, 19.4%; cruroplasty: n = 19, 39.6%, p = 0.059). Among the entire study population, only one patient who underwent full revision experienced an intra-operative complication, which was an esophagotomy that was primarily repaired.

**Table 2:** Comparison of intra-operative characteristics between the full revision and cruroplasty groups

	Full revision (n = 93)	Cruroplasty alone (n = 48)	p-value
Op length, hours	129 (84, 181)	138 (96, 194)	0.349
Intra-op HH size			0.340
Small (< 2 cm)	5 (10.6%)	6 (8.2%)	
Medium (2-5 cm)	11 (23.4%)	4 (12.1%)	
Large (≥ 5 cm)	31 (66.1%)	23 (69.7%)	
Mesh			< 0.001*
None	67 (72.0%)	16 (33.3%)	
Vicryl	8 (8.6%)	13 (27.1%)	
Biosynthetic	18 (19.4%)	19 (39.6%)	
Intraop Complications?	1 (1.1%)	0 (0.0%)	> 0.999

Continuous data expressed as median (interquartile range); categorical data represented as n (%)

^*^p < 0.005

### Post-operative outcomes

Following surgery, patients who underwent full revision had a similar number of 30-day complications compared to patients who received cruroplasty alone (16.1% vs 25.0%, p = 0.285). There was no difference in rates of emergency department (ED) visits, readmissions, reoperations, or endoscopic interventions between the groups.

Mean follow-up time was 1.7 (± 1.4) years for full revision patients and 1.5 (± 1.1) years for cruroplasty alone patients. The most common postop symptom was dysphagia (n = 17, 12.1%), followed by gas bloat (n = 15, 10.6%). There was no significant difference between groups in terms of post-operative symptoms (Table 3). Both groups of patients had similar rates of post-operative PPI use. Additionally, both groups had similar GERD-HRQL scores post-operatively (full revision: 9.6 ± 10.2; cruroplasty: 8.9 ± 11.2, p = 0.829).

**Table 3:** Comparison of post-operative outcomes between the full revision and cruroplasty groups

	Full revision (n = 93)	Cruroplasty alone (n = 48)	p-value
30-day complications	15 (16.1%)	12 (25.0%)	0.285
ED Visit	1 (1.1%)	3 (6.3%)	0.114
Readmission	1 (1.1%)	2 (4.2%)	0.267
Reoperation	3 (3.2%)	1 (2.1%)	> 0.999
Endoscopic Interventions	14 (15.1%)	2 (4.2%)	0.090
Post-op PPI	9 (31.0%)	10 (43.5%)	0.397
Post-op Symptoms			
Dysphagia	10 (27.8%)	7 (21.9%)	0.780
Heartburn	6 (16.7%)	7 (21.2%)	0.761
Regurgitation	5 (14.3%)	5 (17.9%)	0.740
Cough	3 (9.1%)	3 (10.7%)	> 0.999
Hoarseness	1 (3.0%)	2 (7.1%)	0.589
Gas bloat	9 (27.3%)	6 (23.1%)	0.771
GERD-HRQL	9.6 ± 10.2	8.9 ± 11.2	0.829
HH recurrence (HH ≥ 2 cm)	11 (11.8%)	5 (10.4%)	> 0.999

Continuous data expressed as mean ± standard deviation; categorical data represented as n (%)

^*^p < 0.005

In total, 16 (11.3%) patients experienced a subsequent hiatal hernia recurrence. Of these, 11 (11.8%) occurred in the full revision group and 5 (10.4%) occurred in the cruroplasty alone group (p = 0.362). The mean subsequent recurrent hiatal hernia size for full revision patients and cruroplasty alone patients was 3.3 ± 0.6 cm and 4.0 ± 1.4 cm, respectively. Patients who had mesh placed during their revisional surgery had similar rates of recurrence compared to those who did not receive mesh (14.6% vs 14.5%, p = 0.520). Of the patients who had a subsequent recurrence, 3 (27.3%) full revision patients and 1 (20.0%) cruroplasty alone patient had an additional revisional procedure (p > 0.999).

## Discussion

While recurrence rates following hiatal hernia repair remain as high as 60% in 10 years, data regarding the optimal revisional technique remains limited [6]. Previously, standard practice for the management of recurrent hiatal hernias involved full revision with takedown and redo of the prior fundoplication in addition to cruroplasty. However, studies have shown that many recurrent hiatal hernias involved transdiaphragmatic herniation of an intact fundoplication [11, 15]. As such, our institution’s practice is to perform an intraoperative EGD following crural closure. Patients with a Hill Grade of I, indicating an intact fundoplication or MSA device, will undergo cruroplasty alone. Patients with a Hill Grade of II or greater will undergo full revision, with redo fundoplication or MSA. In this study, we evaluated outcomes for patients undergoing cruroplasty alone, with or without mesh, compared to patients undergoing cruroplasty with fundoplication revision. This study demonstrated similar outcomes between the two groups in terms of quality of life, peri-operative outcomes, and recurrence rates, suggesting that cruroplastly alone may be sufficient.

Our study found no difference in operative time between patients undergoing full revision and patients undergoing cruroplasty alone. This may be in part due to the inclusion of patients undergoing MSA in the full revision group, as MSA device placement requires shorter operative times compared to fundoplication [16, 17]. However previous data also demonstrated no difference in operative times between cruroplasty alone and redo fundoplication, suggesting that crural dissection and lysis of adhesions are the most difficult components of revisional surgery [12]. There was no difference in the rates of intraoperative complications between groups, but the incidence of intraoperative complications overall was very low making statistical analysis difficult.

While there was minimal difference in preoperative characteristics between both groups of patients, one notable difference was that cruroplasty alone patients had larger recurrent hiatal hernias compared to the full revision patients. It is not entirely clear why this was the case but may have to do with the inclusion of patients undergoing MSA in the full revision group as initial indications for MSA did not include patients with hiatal hernias larger than 3 cm [18, 19]. Despite the difference in preoperative hiatal hernia size, this study found that patients undergoing cruroplasty alone had similar post-operative outcomes following revision. There was no difference in post-operative PPI use or rates of 30-day complications, readmissions, reoperations, and ED visits between groups. Both groups of patients reported similar post-operative symptoms and GERD-HRQL scores, suggesting comparable satisfaction and quality of life.

Patients who received cruroplasty alone had similar subsequent recurrence rates compared to patients who received full revisions. In this study, cruroplasty alone patients underwent mesh placement significantly more frequently than full revision patients. While some studies suggest the use of mesh during cruroplasty may reduce the risk of recurrence, the use of mesh reinforcement during hiatal hernia repair is controversial [8, 20, 21]. Our data did not find any association between the use of mesh and hiatal hernia recurrence.

There are several limitations to this study. Given its retrospective nature and variation in patient follow-up, there is potential for selection and information bias. Particularly, patients with only short-term follow-up may fail to capture potential benefits or complications which may take longer to evolve. Furthermore, this study analyzed a small sample size of patients, making some statistical analysis difficult. Additional research comparing larger patient populations should be performed to confirm the results of this study.

## Conclusions

This study found no significant difference in short term outcomes, quality of life, and recurrence rates between patients undergoing cruroplasty alone versus cruroplasty and fundoplication revision for recurrent hiatal hernias. This suggests that patients with recurrent hiatal hernias found to have an intact fundoplication or MSA device on intraoperative EGD following crural repair can avoid unnecessary revision and can be safely managed with cruroplasty alone.
